# Electron-Deficient Zn-N_6_ Configuration Enabling Polymeric Carbon Nitride for Visible-Light Photocatalytic Overall Water Splitting

**DOI:** 10.1007/s40820-022-00962-x

**Published:** 2022-11-14

**Authors:** Daming Zhao, Yiqing Wang, Chung-Li Dong, Fanqi Meng, Yu-Cheng Huang, Qinghua Zhang, Lin Gu, Lan Liu, Shaohua Shen

**Affiliations:** 1 International Research Center for Renewable Energy, State Key Laboratory of Multiphase Flow in Power Engineering, Xi’an Jiaotong University, Xi’an, 710049 People’s Republic of China; 2 State Key Laboratory of Rare Earth Resource Utilization, Changchun Institute of Applied Chemistry, Chinese Academy of Sciences, Changchun, 130022 People’s Republic of China; 3 Department of Physics, Tamkang University, New Taipei City, 25137 Taiwan, People’s Republic of China; 4 Beijing National Laboratory for Condensed Matter Physics, Institute of Physics, Chinese Academy of Sciences, Beijing, 100190 People’s Republic of China

**Keywords:** Zn single atoms, Polymeric carbon nitride, Overall water splitting, Photocatalysis

## Abstract

Despite of suitable band structures for harvesting solar light and driving water redox reactions, polymeric carbon nitride (PCN) has suffered from poor charge transfer ability and sluggish surface reaction kinetics, which limit its photocatalytic activity for water splitting. Herein, atomically dispersed Zn-coordinated three-dimensional (3D) sponge-like PCN (Zn-PCN) is synthesized through a novel intermediate coordination strategy. Advanced characterizations and theoretical calculations well evidence that Zn single atoms are coordinated and stabilized on PCN in the form of Zn-N_6_ configuration featured with an electron-deficient state. Such an electronic configuration has been demonstrated contributive to promoted electron excitation, accelerated charge separation and transfer as well as reduced water redox barriers. Further benefited from the abundant surface active sites derived from the 3D porous structure, Zn-PCN realizes visible-light photocatalysis for overall water splitting with H_2_ and O_2_ simultaneously evolved at a stoichiometric ratio of 2:1. This work brings new insights into the design of novel single-atom photocatalysts by deepening the understanding of electronic configurations and reactive sites favorable to excellent photocatalysis for water splitting and related solar energy conversion reactions.

**Supplementary Information:**

The online version contains supplementary material available at 10.1007/s40820-022-00962-x.

## Highlights

- Atomically dispersed Zn-anchored 3D sponge-like polymeric carbon nitride (Zn-PCN) characteristic of a unique Zn-N_6_ electron-deficient configuration is synthesized via an intermediate coordination strategy.
- The electron-deficient Zn-N_6_ configuration contributes to enhanced electron excitation, accelerated charge separation and transfer as well as reduced overpotentials of water redox reactions.
- The obtained Zn-PCN realizes photocatalytic overall water splitting to stoichiometrically produce H_2_ and O_2_ with good durability under visible light.

**Supplementary Information:**

The online version contains supplementary material available at 10.1007/s40820-022-00962-x.

## Introduction

Spontaneously solar-driven water splitting into H_2_ and O_2_ via artificial photosynthesis is very appealing for addressing the dilemma from fossil fuels and environmental issues [1, 2]. Extensive efforts have been made in the past few decades to develop various semiconducting materials as photocatalysts, mainly including metal oxides (*e.g.*, TiO_2_, Fe_2_O_3_, WO_3_, BiVO_4_, SrTiO_3_), metal sulfides (*e.g.*, CdS, Cd_1-*x*_Zn_*x*_S, CuInS_2_, CuZnSnS_4_) and metal (oxy)nitrides (*e.g.*, Ta_3_N_5_, TaON, GaN/ZnO, LaTiO_2_N) [3, 4]. Recently, polymeric semiconductors with adjustable molecular structures and tunable band structures have been documented as the promising alternatives to inorganic photocatalysts for water splitting [5–7]. Typically, as an organic semiconductor mainly composed of carbon and nitrogen, polymeric carbon nitride (PCN) with a two-dimensional (2D) conjugated structure has inspired extensive research in photocatalysis for H_2_ and/or O_2_ production, owing to its abundant precursors, environmental friendliness, chemical stability and proper band potentials for water redox reactions [8–11], since its pioneering demonstration by Wang et al*.* [12]. However, the intrinsic drawbacks, especially the high charge-carrier recombination rate and the sluggish surface reaction kinetics, largely restrict the photocatalytic efficiency of PCN [8]. To conquer these limitations, a variety of strategies including heteroatoms doping, defect engineering, heterojunction constructing, morphology design, etc*.,* [13–18] have emerged to modify PCN for photocatalytic water splitting, with performances continually hitting record highs.

As a hot spot of the current research in catalysis, metal single-atom catalysts with nearly 100% atomic utilization and superb catalytic properties have received increasing attentions in the fields of thermocatalysis, electrocatalysis and photocatalysis [19–23]. Given the large surface free energy induced by the extremely small size, a support that could chemically interact and then highly disperse single atoms is always desired to prohibit the aggregation and ensure the stability of single-atom catalysts. With abundant electron-rich pyridinic nitrogen atoms in the tri-s-triazine units, PCN has been believed as an ideal support for stabilizing single atoms, by providing sufficient coordination sites to anchor metal atoms via the formed strong covalent metal-N bonds [9, 24]. In turn, the physicochemical property of PCN support would be concomitantly changed with the aid of the coordinated single atoms. Thanks to the fine-tunable electronic structure of PCN and the high catalytic activity of single-atom active sites, PCN-based metal single-atom photocatalysts have shown great potentials in photocatalytic water splitting, with charge transfer processes promoted and surface reaction kinetics accelerated [25–29]. For instance, Guo group developed a PH_3_-assisted thermolysis strategy to create a phosphorus-coordinated noble metal single-atom coordination on PCN [25]. With an electron-rich feature in the MP_2_ (M = Ru, Rh, Pd) coordination contributing to reduced reaction barriers, the obtained MP_2_/PCN exhibited excellent visible-light photocatalytic H_2_ production activity and stability. Bi and co-workers achieved a significantly improved H_2_ production activity by anchoring single-atom Pt onto PCN via photochemical deposition, and elucidated the separated reduction and oxidation reactions sites by discovering the dynamic variations of Pt–N bond/Pt^0^ species and the corresponding C-N bond/C = N bond in single-atom Pt/PCN during the photocatalytic water splitting process using synchronous illumination X-ray photoelectron spectroscopy [26]. Shi et al*.* introduced Ni single-atom sites onto PCN via a freezing-deposition method and precisely modulated the oxidation state of Ni into the intermediate state with a Ni^2+^/Ni^0^ ratio of 2, which brought abundant unpaired *d*-electrons in the Ni/PCN photocatalyst [27]. Such an electronic configuration resulted in promoted light response, conductivity, charge separation and mobility, thus enhancing the photocatalytic water reduction performance. With significant advances achieved for PCN-based metal single-atom photocatalysts, the insightful understanding of structure–activity relationship is still essential for the development and design of novel single-atom photocatalysts toward efficient solar-hydrogen conversion.

Herein, atomically dispersed Zn-coordinated three-dimensional (3D) sponge-like PCN (Zn-PCN) was synthesized via a novel intermediate coordination strategy, with single-atom loading content up to 4.79% and specific surface area reaching 224.99 m^2^ g^−1^. In comparison with bulk PCN (BCN), the obtained Zn-PCN achieves significantly improved photocatalytic H_2_ production activity from water splitting in the presence of sacrificial agent. Surprisingly, Zn-PCN is capable of photocatalytic overall water splitting under visible light, producing H_2_ and O_2_ at a stoichiometric ratio of 2:1, with H_2_ production rate reaching 35.2 μmol h^−1^ g^−1^. Along with the 3D porous structure beneficial to abundant surface active sites, experimental and calculation results demonstrate that Zn single atoms are steadily anchored on PCN via a unique Zn-N_6_ electron-deficient coordination, which induces a midgap energy level, increases the carrier concentration, and strengthens the charge polarization, contributing to enhanced electron excitation, accelerated charge separation and transfer as well as reduced overpotentials of water redox reactions. This work provides a new idea for the design of novel single-atom photocatalysts toward solar water splitting with in-depth understanding of the structure–activity relationship from the level of atomic coordination and electronic configuration.

## Experimental Section

### Materials

Melamine (C_3_H_6_N_6_, ≥ 99.0%), hydrochloric acid (HCl, ~ 36.0–38.0 wt%), sodium sulfate (Na_2_SO_4_, ≥ 99.0%), and triethanolamine (TEOA, analytical reagent) were purchased from Sinopharm Chemical Reagent Co., Ltd. Zinc carbonate (ZnCO_3_, 97%) and chloroplatinic acid hexahydrate (H_2_PtCl_6_·6H_2_O, ACS reagent) were purchased from Alfa Aesar. Heavy-oxygen water (H_2_^18^O, 99%) was purchased from 3A chemicals Co., Ltd. Ultrahigh-purity argon (Ar, ≥ 99.999%) was provided by Shaanxi Xinkang Medical Oxygen Co., Ltd. All the materials were used as received without further purification. Deionized water, with a resistivity of 18.25 MΩ cm, was used throughout the experiments.

### Synthesis of Bulk PCN

Bulk PCN (BCN) was synthesized by thermal polymerization of melamine. Typically, 2 g of melamine was put into a covered alumina crucible and calcined at 520 °C in Ar for 4 h with a ramping rate of 5 °C min^−1^. After naturally cooled to room temperature, the yellow BCN was collected and ground into powder.

### Synthesis of Atomically Dispersed Zn-PCN

Atomically dispersed Zn-coordinated 3D sponge-like PCN was synthesized through an intermediate coordination method. In detail, 2 g of melamine was mixed with a certain amount of ZnCO_3_ (1, 2, 4 g) and then calcined at 400 °C in Ar for 2 h with a ramping rate of 5 °C min^−1^. After naturally cooled to room temperature, the resultant solid mixtures were added into excessive HCl aqueous solution (1.0 mol L^−1^) and vigorously stirred for 12 h, followed by washing with deionized water until the supernatant was neutral. After freeze drying (24 h), the obtained powders were calcined at 520 °C in Ar for 4 h with a ramping rate of 5 °C min^−1^. The final products were denoted as Zn-PCN(*x*) (*x* represents the weight contents of Zn single atoms, *x* = 2.09%, 3.63%, 4.79%).

### Characterization

The weight contents of zinc were measured by an inductively coupled plasma mass spectrometer (NexION 350D, PerkinElmer). Transmission electron microscopy (TEM) images were recorded on a FEI Tecnai G2 F30 S-Twin transmission electron microscope at an accelerating voltage of 300 kV. An OXFORDMAX-80 energy-dispersive X-ray detector, mounted in this microscope, was used to conduct the elemental analysis. The high-angle annular dark-field scanning transmission electron microscopy (HAADF-STEM) was performed on a JEOL JEM-ARM200F transmission electron microscope operated at 200 kV and equipped with double spherical aberration correctors. Scanning electron microscopy (SEM) images were obtained by a JEOL JSM-7800F field-emission scanning electron microscope. N_2_ adsorption–desorption isotherms were conducted using a surface area and porosity analyzer (ASAP 2020, Micromeritics) at 77 K after degassing the samples at 150 °C for 4 h. The specific surface area and pore volume were determined by the Brunauer–Emmett–Teller method. The pore size distribution was calculated using the Barrett–Joyner–Halenda method. X-ray diffraction (XRD) patterns were collected on a PANalytical X’pert Pro MPD diffractometer operated at 40 kV and 40 mA using Ni-filtered Cu Kα irradiation (wavelength = 1.5406 Å). Fourier transform infrared (FTIR) spectra were recorded using a Bruker Vertex 70 FTIR spectrometer using the KBr pellet technique. The amount of sample used for each FTIR spectrum was kept at around 1 mg. X-ray photoelectron spectroscopy (XPS) data were obtained on a Kratos Axis-Ultra DLD spectrometer with a monochromatized Al Kα line source (150 W). All the binding energies were referenced to the C 1*s* peak at 284.8 eV. Ultraviolet–visible-near infrared diffuse reflectance spectra were recorded on a Cary Series ultraviolet–visible-near infrared spectrophotometer (Agilent Technologies). The surface photovoltage (SPV) spectra were obtained on a self-made measurement system, equipped with a 500 W Xe lamp (CHF-XM500, Beijing PerfectLight), a grating monochromator (Omni-λ3007, Zolix), a lock-in amplifier (SR830-DSP) with an optical chopper (SR540) and a photovoltaic cell. The steady-state photoluminescence (PL) emission spectra and time-resolved transient PL decay spectra were measured at room temperature using a PTI QM-4 fluorescence spectrophotometer. Considering the existence of three radiative lifetimes, the PL decay curve of PCN was triexponentially fitted using equation [16]:

1 $$I\left( t \right) = A_{1} e^{{ - t/\tau_{1} }} + A_{2} e^{{ - t/\tau_{2} }} + A_{3} e^{{ - t/\tau_{3} }}$$

2 $$\tau_{{{\text{avg}}}} = \frac{{\left( {A_{1} \tau_{1}^{2} + A_{2} \tau_{2}^{2} + A_{3} \tau_{3}^{2} } \right)}}{{\left( {A_{1} \tau_{1} + A_{2} \tau_{2} + A_{3} \tau_{3} } \right)}}$$

where *A*_1_, *A*_2_ and *A*_3_ represent the normalized amplitudes of each decay component, respectively, *τ*_1_, *τ*_2_ and *τ*_3_ are values of the lifetime components, respectively, *τ*_avg_ is average lifetime of photogenerated charge carriers.

### Synchrotron-Based X-ray Spectroscopic Measurements and Data Analysis

The synchrotron-based X-ray spectroscopic measurements at the C, N and Zn K-edge were carried out at beamline BL20A, at the National Synchrotron Radiation Research Center. The acquired EXAFS data were processed according to the standard procedures using the ATHENA module implemented in the IFEFFIT software packages [25]. The *k*^3^-weighted χ(*k*) data in the *k*-space ranging from 2.893 to 10.99 Å^−1^ were Fourier transformed to real (*R*) space using Hanning windows (d*k* = 1.0 Å^−1^) to separate the EXAFS contributions from different coordination shells. To obtain the detailed structural parameters around Zn atom in the as-prepared samples, the quantitative curve fittings were performed for the Fourier transformed *k*^3^χ(*k*) in the *R*-space using the ARTEMIS module of IFEFFIT. Effective backscattering amplitudes F(*k*) and phase shifts Φ(*k*) of all fitting paths were calculated by the ab initio code FEFF8.0. During the fitting analysis, the amplitude reduction factor S_0_^2^ was fixed to the best-fit value of 0.8011, which was determined from fitting the reference sample of Zn foil. To fit the data of Zn-PCN, the Zn-N coordination number, interatomic distance (*R*), Debye–Waller factor (*σ*^2^) and inner potential shift (Δ*E*_0_) were allowed to vary. The obtained structural parameters are summarized in Table S2.

### Density Functional Theory Calculations

First-principles density functional theory (DFT) calculations were performed using the CASTEP code [30]. The exchange–correlation effects were treated in generalized gradient approximation (GGA) with the Perdew–Burke–Ernzerhof (PBE) potential. The interaction between ionic core and valence electrons was simulated by the ultrasoft pseudopotentials. The kinetic energy cut-off was chosen to be 500 eV. Brillouin zone integration was sampled with 3 × 3 × 3 and 3 × 3 × 1 Monkhorst–Pack mesh K-points for BCN and Zn-PCN calculation, respectively. The slab thickness for three-layer structure models is 6.54 Å. The slabs were set by a vacuum region of 20 Å. The adsorbates and the top layers of the surfaces are allowed to fully relax and optimize until convergence to 10^–5^ eV in total energy and 0.01 eV Å^−1^ in the forces.

### Photoelectrochemical Measurements

The photoelectrochemical properties were measured on an electrochemical workstation (CHI760D) in a three-electrode cell system under light irradiation, in which platinum foil and Ag/AgCl electrode were used as the counter and reference electrodes, respectively. The as-prepared photocatalysts were electrodeposited on fluoride tin oxide (FTO) glass substrates and used as the working electrodes. In detail, 40 mg of photocatalysts were ultrasonically dispersed in 50-mL acetone solution of I_2_ (10 mg). One piece of clean FTO glass (1 × 1.5 cm^2^) and one platinum foil in parallel, immersed in the above dispersion, were applied with a potential of − 5 V for 5 min. The immersed area of the FTO glass was fixed at 1 × 1 cm^2^. Then, the deposited electrode was dried at 120 °C for 30 min to remove the I_2_ residue. 0.5 M Ar-saturated Na_2_SO_4_ aqueous solution was used as the electrolyte. A 500 W Xe lamp (CHF-XM500, Beijing PerfectLight) with adjustable power settings through an air mass 1.5 global (AM1.5) filter was used as light source (100 mW cm^−2^). The illumination area of working electrode was fixed at 0.785 cm^2^. The transient photocurrent response with an interval of 10 s on/off switch was measured at an applied potential of 0.2 V versus Ag/AgCl. Electrochemical impedance spectra (EIS) were recorded at an applied potential of 0.8 V versus Ag/AgCl. The frequency varied from 10 kHz to 0.1 Hz, and the amplitude of the sinusoidal wave was set at 5 mV. The Mott–Schottky plots were measured with 10 mV amplitude at different frequencies (1000, 2000 and 3000 Hz).

### Photocatalytic Measurements

Photocatalytic H_2_ production reactions were performed in a hermetic Pyrex reactor. In a typical reaction, 10 mg of photocatalysts were dispersed in 100-mL aqueous solution of TEOA (10 vol%) using a magnetic stirrer with constant rotational velocity. 1 wt% Pt as cocatalyst was photodeposited on the photocatalyst from the precursor of H_2_PtCl_6_·6H_2_O. Ar was purged through the reactor for 30 min before reaction to remove the residual air. A 300 W Xe lamp (PLS-SXE300, Beijing PerfectLight) with a 420-nm cut-off filter (*λ* > 420 nm) was used as light source to trigger the photocatalytic H_2_ generation. The temperature of the reaction solution was kept at 35 °C via a circulating water pump during the whole experiment. Evolved H_2_ was measured through a gas chromatograph equipped with a thermal conduction detector (5 Å molecular sieve column, Ar as carrier gas). Photocatalytic overall water splitting reactions were conducted under the same photocatalytic reaction condition without any carrier scavenger. Blank experiments revealed no appreciable gas production without irradiation or photocatalysts. Photocatalytic H_2_^18^O (99%) splitting measurements were detected on an Agilent 7890A-5975C gas chromatograph–mass spectrometer.

## Results and Discussion

### Catalyst Synthesis and Morphology

**Fig. 1:**
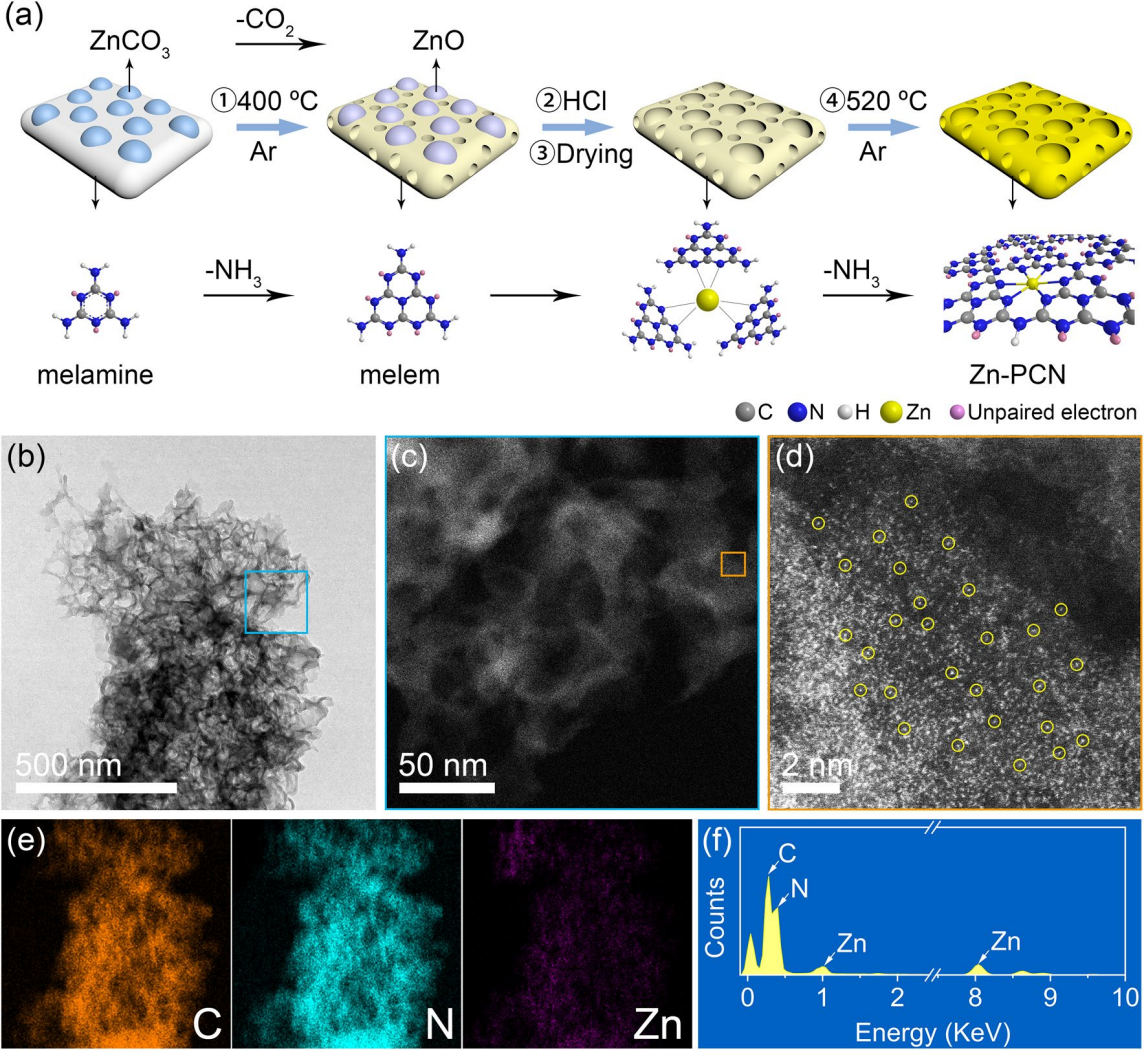
**a** Schematic illustration of the synthesis of Zn-PCN **b** TEM image of Zn-PCN. Representative HAADF-STEM images of Zn-PCN at **c** low and **d** high magnifications zoomed in at the square regions in **b** and **c**, respectively. **e** Elemental mappings of C, N and Zn acquired from **b**. **f** EDS spectrum acquired from **b**

Zn-PCN was synthesized through an intermediate coordination method by using melamine as the precursor of PCN, and ZnCO_3_ as zinc sources and also the template for 3D sponge-like structure (see Experimental Section for details). As shown in Fig. 1a, through the first-step calcination, a mixture of melem monomer and ZnO was produced from melamine and ZnCO_3_. With ZnO leached by hydrochloric acid (HCl) solution, the dried mixture, in which residual Zn^2+^ species might coordinate with the pyridinic nitrogen atoms of melem monomer [9, 31], was then calcined again to obtain the Zn-PCN products via the polymerization of melem intermediates with the coordinated Zn single atoms. By changing the amounts of ZnCO_3_ precursor, the weight contents (*x*) of Zn single atoms in Zn-PCN(*x*) could be readily tuned (*x* = 2.09%, 3.63%, 4.79%), as quantified by inductively coupled plasma mass spectroscopy. As compared to BCN synthesized by direct thermal polymerization of melamine (Fig. S1a-b), the obtained Zn-PCN presents a unique 3D sponge-like morphology (Figs. S1c-d), with the produced ZnO species acting as hard templates and the released NH_3_ and CO_2_ gases acting as soft templates during the first-step calcination. As learned from the N_2_ adsorption–desorption measurements (Fig. S2 and Table S1), Zn-PCN owns a typical mesoporous structure with average pore diameter of ~ 30 nm. Depending on the increasing amounts of ZnCO_3_ precursor (*i.e.*, Zn loading contents), the specific surface area gradually increases, reaching 224.99 m^2^ g^−1^ as high for Zn-PCN(4.79%), which is almost 24 times that for BCN (9.46 m^2^ g^−1^). Such a well-developed 3D porous structure with increased specific surface area and inhibited agglomeration could be confirmed by the weakened XRD diffraction peaks (Fig. S3) [15, 32], which would provide abundant surface active sites for accelerating photocatalytic reaction [33, 34]. With nanoparticle and clusters of ZnO or other Zn-based compounds hardly identified in TEM image (Fig. 1b) and XRD patterns (Fig. S3), the high dispersion of Zn single atoms on PCN could be further evidenced from the spherical aberration-corrected high-angle annular dark-field scanning transmission electron microscopy (HAADF-STEM) images (Fig. 1c, d). One could easily discover densely and evenly isolated bright spots with sizes < 0.2 nm (highlighted by yellow circles) that could be assigned to the Zn atoms heavier than the non-metal atoms such as N and C in PCN [25–29], which unambiguously confirms the good dispersion of Zn atoms with high density in Zn-PCN. With the existence of Zn element further affirmed by energy-dispersive X-ray spectroscopy (EDS) spectrum, elemental mappings again reveal the homogeneous dispersion of Zn atoms along with elemental C and N in Zn-PCN (Fig. 1e, f). These microscopy observations provide solid evidences for the high dispersion of Zn single atoms anchored on the PCN support, with the molecular structural characteristics of both tri-s-triazine ring and trigonal C-N(-C)-C/bridging C-NH-C units well-maintained during the intermediate coordination and polymerization process for the introduction of Zn single atoms (Fig. S4).

### Structure Determination

**Fig. 2:**
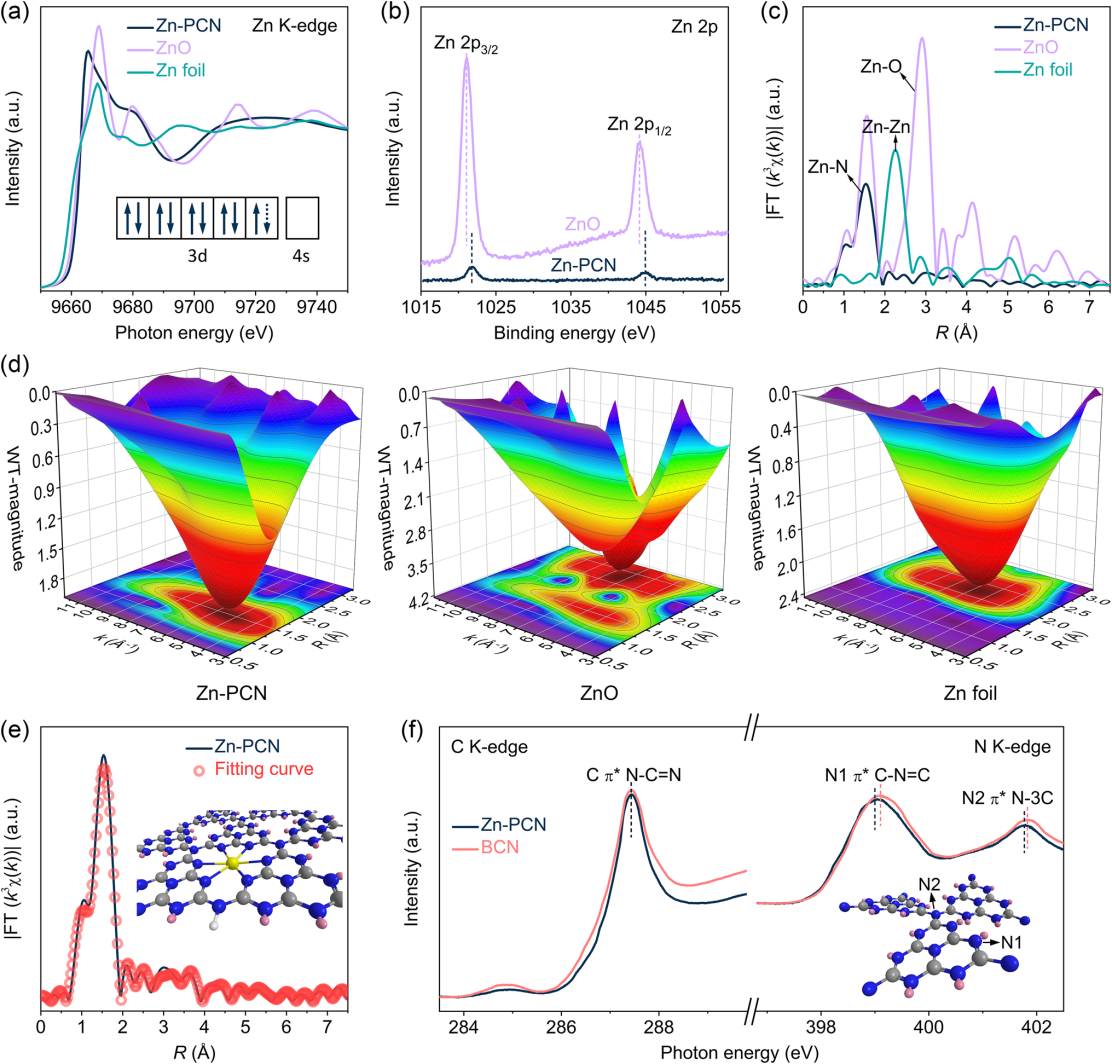
**a** Zn K-edge XANES spectra of Zn-PCN, ZnO and Zn foil. The inset in **a** shows the outer electron configuration of Zn single atom in Zn-PCN. **b** High-resolution Zn 2*p* XPS spectra of Zn-PCN and ZnO. **c** Zn K-edge *k*^3^-weighted FT-EXAFS spectra of Zn-PCN, ZnO and Zn foil. **d** WT-EXAFS spectra of Zn-PCN, ZnO and Zn foil. **e** Fitting curve of FT-EXAFS for Zn-PCN. The inset in **e** shows the structure model of Zn-PCN with one Zn atom coordinated by six N atoms. The gray, blue, white, yellow and pink spheres represent the C, N, H, Zn atoms and the unpaired electrons, respectively. **f** C K-edge and N K-edge XANES spectra of BCN and Zn-PCN. The inset in **f** illustrates the two types of N atoms in PCN network

The chemical state and coordination environment of Zn single atoms in Zn-PCN were determined by synchrotron-based X-ray spectroscopic measurements collected at the Zn K-edge of Zn-PCN and some references (ZnO and Zn foil). As shown in the Zn K-edge X-ray absorption near-edge structure (XANES) spectra (Fig. 2a), the absorption threshold for Zn-PCN is significantly higher than that for Zn foil and even higher than that for ZnO, indicating that Zn single atoms in Zn-PCN exist at an oxidation state higher than that of Zn^2+^ and hold more unoccupied states than Zn^2+^ [29, 31], as further confirmed by that the characteristic peaks of Zn 2*p*_3/2_ and Zn 2*p*_1/2_ levels for Zn-PCN shift to higher binding energies as compared to ZnO in the Zn 2*p* XPS spectra (Fig. 2b) [35]. Considering that Zn^2+^ has a 3*d*^10^ electron configuration, Zn single atoms should possess a partially vacant 3*d* orbital (inset in Fig. 2a), attributed to the electron transfer from Zn single atoms to PCN support. Note the two O 1*s* XPS signals at 530.2 and 531.8 eV for ZnO as reference (Fig. S5), assigned to the lattice oxygen in wurtzite structure and defective oxygen, respectively [36]. Zn-PCN only displays a weak O 1*s* XPS signal assigned to absorbed oxygen (532.1 eV) [37], excluding the Zn–O interaction in Zn-PCN. In the Fourier transform extended X-ray absorption fine structure (FT-EXAFS) spectra (Fig. 2c), without signals detected for Zn–O interaction (~ 2.9 Å) or Zn-Zn interaction (~ 2.3 Å), only a distinct peak attributed to Zn-N interaction (~ 1.5 Å) could be observed for Zn-PCN [38, 39], suggesting that Zn single atoms should be coordinated with the unsaturated N sites in the tri-s-triazine units of Zn-PCN with an electron-deficient state. As further revealed by the wavelet transforms (WT) of EXAFS (Fig. 2d), rather than the peaks at *k* =  ~ 8.0 Å^−1^ observed for ZnO and at *k* =  ~ 7.0 Å^−1^ for metallic Zn, Zn-PCN presents a single peak at *k* =  ~ 5.5 Å^−1^ in the contour plot, which again corroborates the Zn-N coordination in Zn-PCN. With the quantitative structural parameters of Zn single atoms in Zn-PCN confirmed by the fitted FT-EXAFS curve, it is convincing that Zn coordinated by six pyridinic N (*i.e.*, Zn-N_6_) should be the dominant coordination structure in Zn-PCN (Fig. 2e and Table S2).

In addition to the characterization of Zn single atoms, XANES measurements at C K-edge and N K-edge were also performed to further elucidate the local atomic and electronic structure changes in Zn-PCN versus BCN. As shown in Fig. 2f, the C K-edge peak at 287.4 eV is associated with the electron transition from C 1*s* to 2p π* orbital related to N–C = N *sp*^2^ hybridized states in BCN; the N K-edge peaks at 399.1 and 401.8 eV correspond to the N1 site of C-N = C coordination structure in a tri-s-triazine unit and the N2 site of N-3C bridging structure connecting with three tri-s-triazine units, respectively [32]. One could observe that both the C K-edge and N K-edge peaks of Zn-PCN shift toward photon energy with lower peak intensity as compared to those of BCN (Fig. 2f), meaning the decreased π* characteristics of Zn-PCN due to the chemical coordination between tri-s-triazine units and the anchored Zn single atoms [16], as evidenced in the Zn K-edge XANES spectra (Fig. 2a). Thereafter, the less unoccupied 2*p* states at tri-s-triazine units of Zn-PCN could generally accelerate the electron transfer from Zn single atoms to tri-s-triazine units and thus benefits photocatalysis [31].

### Density Functional Theory Calculations

**Fig. 3:**
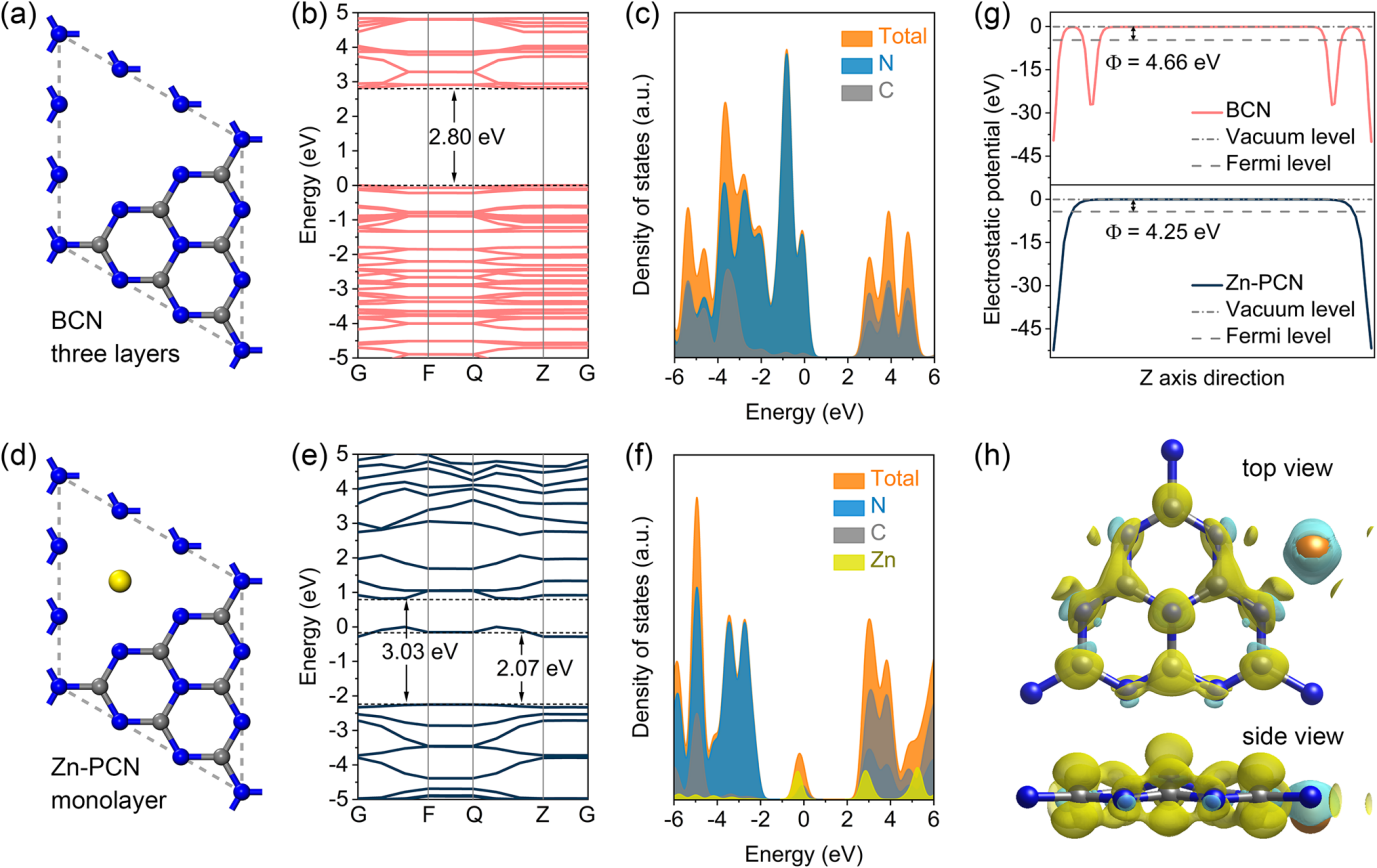
Structure models of **a** BCN and **d** Zn-PCN. **b**, **e** Calculated band structures and **c**, **f** corresponding DOS of BCN and Zn-PCN, respectively. **g** Electrostatic potentials of BCN (002) plane and Zn-PCN (002) plane. **h** Differential charge density map of Zn-PCN. The iso-surface value is 0.012 e Å^−3^. The yellow and cyan regions represent net electron accumulation and depletion, respectively. The gray, blue and yellow spheres represent the C, N and Zn atoms, respectively

DFT calculations were performed to better understand the electronic configuration evolution of PCN induced by the anchored Zn single atoms. With monolayer and three-layer structure models set for 3D sponge-like Zn-PCN and BCN, respectively, the band structure and density of states (DOS) calculation results demonstrate that both C 2*p* and N 2*p* orbitals contribute to the conduction band (CB), while the valence band (VB) is dominated by N 2*p* orbitals for PCN (Fig. 3a-c), which is well-consistent with previous reports [40]. Upon the introduction of Zn single atoms, Zn 3*d* orbitals greatly contribute to the CB of Zn-PCN, with a new Zn-related midgap energy level appearing at ~ 2.07 eV above the VB (Fig. 3d–f), coinciding with the extended absorption tails seen in the ultraviolet–visible-near infrared diffuse reflectance spectra (UV–vis-NIR DRS, Fig. S6a) as well as the experimentally determined band structures (Fig. S6b–d). Meanwhile, the bandgap of Zn-PCN is widened as compared to BCN (Fig. 3b, e), agreeing with the blueshift in optical absorption edge upon the introduction of Zn single atoms (Fig. S6a), which should be related to the well-developed 3D porous structure inhibiting the agglomeration of Zn-PCN [32]. It should be noted that the different Zn single-atom loading contents do not obviously change the bandgap of Zn-PCN (Fig. S7a-f). However, the high Zn single-atom loading content would give rise to a small work function (Φ) of Zn-PCN (Fig. S7g), with the work function calculated for Zn-PCN (4.25 eV) much smaller than that of BCN (4.66 eV) (Fig. 3g), which suggests the higher carrier concentration, contributing to the better charge transfer capability and the stronger electron excitation in Zn-PCN (Fig. S7h–i) [27, 41]. The differential charge density map (Fig. 3h) witnesses the charge redistribution in Zn-PCN with holes mainly enriched at Zn single atoms and electrons accumulated at the adjacent C and N atoms, further implying the electron-deficient feature of Zn single atoms. Such localized charge distribution would spatially separate the redox active sites from each other, inhibiting the recombination of photogenerated electron–hole pairs and the back reaction of H_2_ and O_2_ evolved at surface [31].

### Electron Excitation and Charge Transfer Properties

**Fig. 4:**
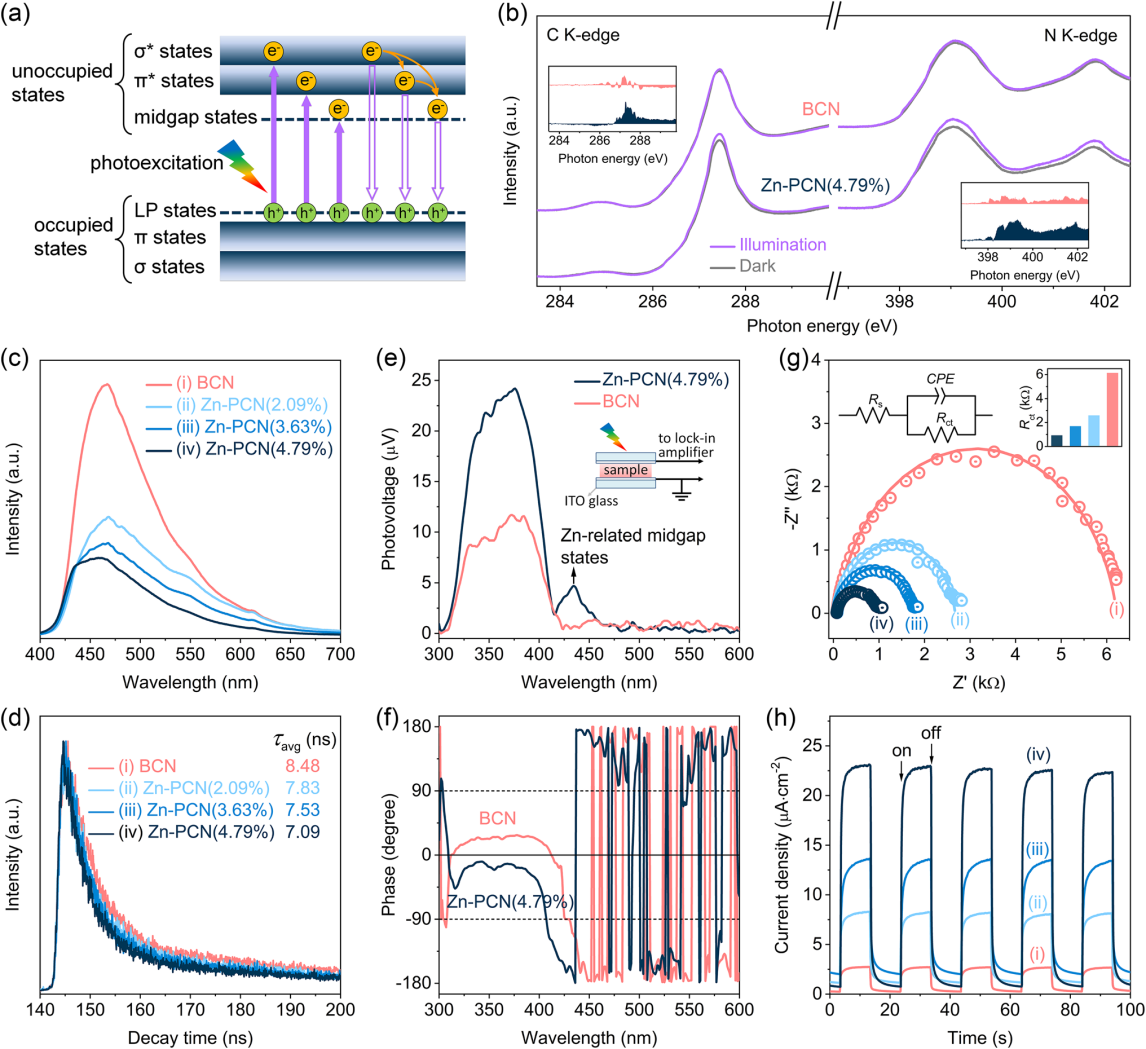
**a** Illustration of the electron transition processes in Zn-PCN. **b** C K-edge and N K-edge XANES spectra of BCN and Zn-PCN(4.79%) with or without illumination. The inset in **b** shows the spectral difference between being in dark and light. **c** Steady-state PL emission spectra and **d** time-resolved transient PL decay spectra of BCN and Zn-PCN. The excitation wavelength for all samples is 337 nm. **e** SPV spectra of BCN and Zn-PCN(4.79%) and **f** corresponding phase spectra. The inset in **e** shows the schematic setup for the SPV measurements. **g** Electrochemical impedance spectra of BCN and Zn-PCN at 0.8 V versus Ag/AgCl in 0.5 M Na_2_SO_4_ aqueous solution under light irradiation. The left inset in **g** is an equal circuit for the catalyst/electrolyte interfaces. *R*_s_ is the electrolyte resistance. *R*_ct_ and *CPE* is the charge transfer resistance from the bulk to the surface of photocatalyst and the constant phase element, respectively. The right inset in **g** presents the corresponding *R*_ct_ values. **h** Transient photocurrent density curves of BCN and Zn-PCN at 0.2 V versus Ag/AgCl in 0.5 M Na_2_SO_4_ aqueous solution under sequential illumination condition with an interval of 10 s on/off switch

Above calculation results indicate that the introduced Zn single atoms could benefit the electron excitation by modulating the electronic configuration in Zn-PCN, which was further explored by the C K-edge and N K-edge XANES spectra recorded both in dark and under illumination to monitor the photoexcited electron transition in Zn-PCN. As previously reported [42], PCN is composed of tri-s-triazine units interconnected with tertiary nitrogen atoms, where C-N *sp*^3^ hybridization constitutes the high-energy *σ* and σ* molecular orbitals, while C-N *sp*^2^ hybridization gives rise to the low-energy *π* bonding and π* antibonding orbitals. In addition, the unpaired electrons on pyridinic N atoms create a lone pairs (LP) orbital above the π bonding orbital, and vacancies or dopants would induce a midgap energy level. These orbitals form the occupied and unoccupied states, respectively (Fig. 4a). It is clear that with the introduction of Zn single atoms, the irradiation-dark intensity variations in both C K-edge and N K-edge spectra are more obvious in Zn-PCN than in BCN (Fig. 4b), supporting the promoted electron excitation from LP states to π* states in the CB of Zn-PCN [43], which should be related to the increased carrier concentration caused by the electron injection from Zn single atoms to PCN [27]. Then, the charge transfer processes in Zn-PCN was investigated by photoluminescence (PL) spectroscopy. As shown in Fig. 4c, a strong PL emission peak centered at ~ 465 nm could be observed for BCN, which is related to the electron transition between π* antibonding orbital and LP orbital [12, 42]. By comparison, Zn-PCN show a dramatical PL quenching (Fig. 4c), implying that the radiative recombination of photogenerated electron–hole pairs in Zn-PCN could be substantially inhibited by the introduced midgap energy level (Fig. 4a) and the strengthened charge polarization (Fig. 3h) by the anchored Zn single atoms [25, 26]. Note that the gradual blueshift in PL emission peak depends on the increasing amounts of Zn single atoms, inferring the widened bandgaps in accordance with the UV–vis-NIR DRS (Fig. S6a). In addition, the time-resolved transient PL decay spectra recorded at the corresponding steady-state emission peaks reveal the shortened average lifetime (*τ*_avg_) of photogenerated charge carriers in Zn-PCN with respect to BCN (Fig. 4d and Table S3), meaning that the Zn single atoms coordinated on PCN would effectively accelerate the charge transfer process [28].

Another solid evidence strongly supporting the improved charge transfer ability triggered by the introduced Zn single atoms could be accounted for by surface photovoltage (SPV) spectra. As shown in Fig. 4e, the SPV signals of both BCN and Zn-PCN are positive, a characteristic of n-type semiconductors, revealing that photogenerated holes transfer to the surface of PCN under illumination [44]. The more positive SPV signal observed for Zn-PCN than BCN indicates that the introduction of Zn single atoms would enable more holes to transfer to the surface of PCN [44]. This Zn single atoms promoted charge-carrier transfer process could be affirmed by the corresponding phase spectra (Fig. 4f), in which the phase changes from the first quadrant for BCN to the fourth quadrant for Zn-PCN [45]. Considering the electron-deficient feature of Zn single-atom sites, more photogenerated holes transfer to Zn single atoms under light irradiation, thus accelerating the charge transfer in Zn-PCN. Moreover, a new SPV signal appears at the wavelength range of 420–450 nm for Zn-PCN (Fig. 4e), again confirming the enhanced electron excitation originated from Zn-related midgap energy level (Fig. 4a) [16].

The charge transfer behavior in Zn-PCN, which was electrodeposited on a conductive substrate as a photoelectrode, was further evaluated by photoelectrochemical measurements. As revealed by the electrochemical impedance spectra of the as-deposited photoelectrodes (Fig. 4g), with Nyquist plots fitted by a typical equivalent circuit model (Table S4 and the inset in Fig. 4g), Zn-PCN exhibits much decreased charge transfer resistances in comparison with BCN, indicating the faster charge transfer processes in Zn-PCN [46], which is in accordance with the above PL and SPV results. These consistent conclusions can also be supported by the photoelectric response tests (Fig. 4h), which were recorded for several on–off cycles of intermittent irradiation. It is clear that the transient photocurrent densities of Zn-PCN are much higher than that of BCN, again providing reasonable evidence for the promoted charge transfer in Zn-PCN for surface water redox reaction under light irradiation [47].

### Photocatalytic Performance for Water Splitting

**Fig. 5:**
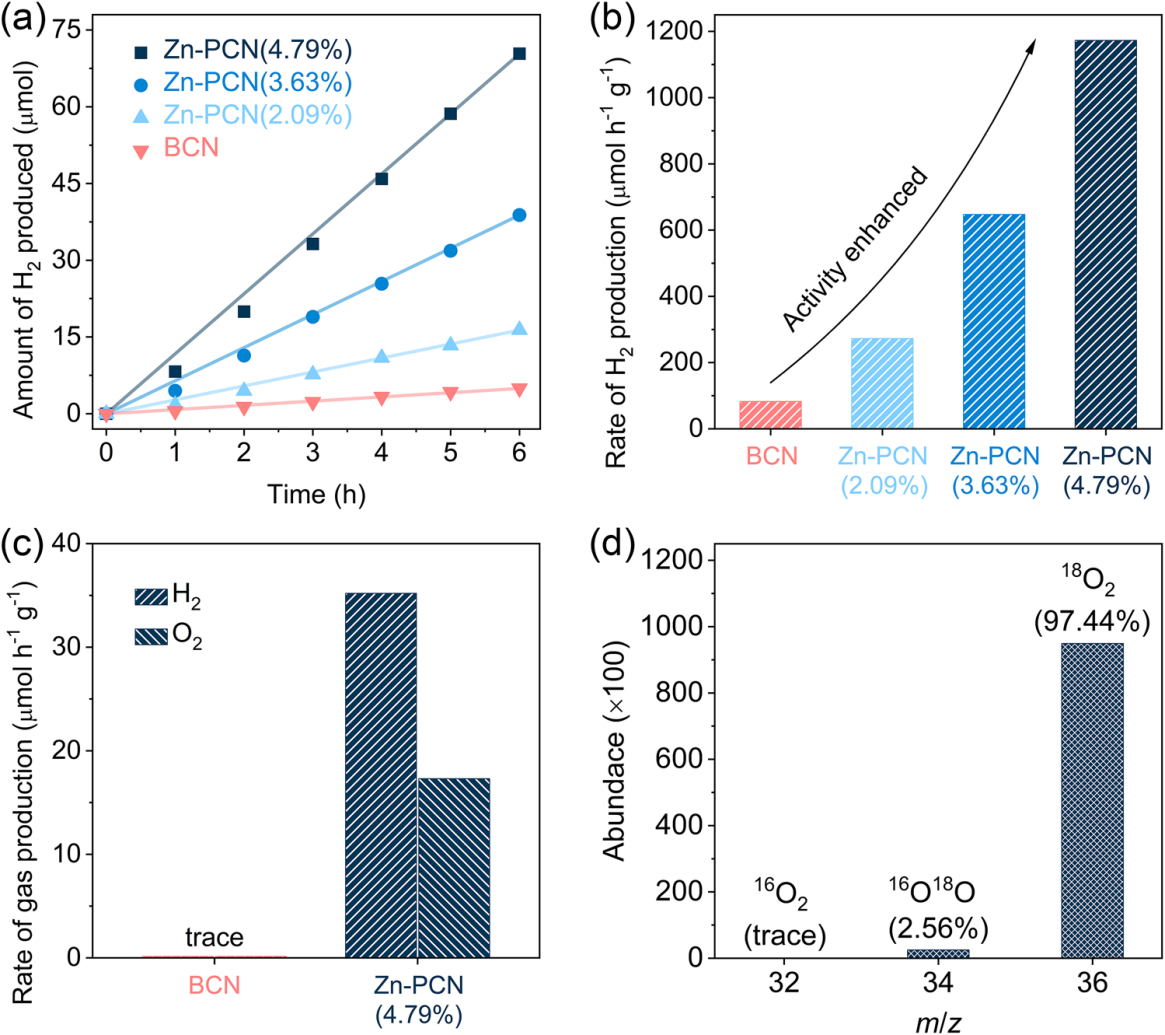
**a** Amounts of H_2_ produced and **b** H_2_ production rates of BCN and Zn-PCN from TEOA aqueous solutions under visible-light irradiation (*λ* > 420 nm). **c** Photocatalytic overall water splitting performance over BCN and Zn-PCN (4.79%) under visible-light irradiation (*λ* > 420 nm). **d** The mass spectrum data of O_2_ evolved from photocatalytic H_2_^18^O (99%) splitting over Zn-PCN (4.79%)

The photocatalytic activities of the as-prepared samples were evaluated under visible-light irradiation (*λ* > 420 nm) for H_2_ production from aqueous solution containing hole scavenger or pure water without any sacrificial agent. With triethanolamine (TEOA) used as hole scavenger, BCN could produce H_2_ at a low rate of only 82.7 µmol h^−1^ g^−1^ (Fig. 5a, b). By comparison, the Zn-PCN samples exhibit gradually increased photocatalytic activity for H_2_ production, depending on the increasing amounts of Zn single atoms. The highest H_2_ production rate could reach 1172.9 µmol h^−1^ g^−1^ for Zn-PCN (4.79%), which is more than 14 times that of BCN (82.7 µmol h^−1^ g^−1^). It is generally accepted that BCN can hardly split pure water under visible light (Fig. 5c). Encouragingly, Zn-PCN(4.79%) is capable of producing H_2_ and O_2_ from pure water at a stoichiometric ratio of 2:1, with H_2_ and O_2_ production rates reaching 35.2 and 17.3 µmol h^−1^ g^−1^, respectively. To illustrate the origin of the O_2_ product, ^18^O isotope-labeled photocatalytic overall water (H_2_^18^O, 99%) splitting measurement was performed over Zn-PCN(4.79%). The molar content of the labeled ^18^O_2_ with *m*/*z* of 36 is detected to be 97.44% (Fig. 5d), which is very close to the theoretical value of 98.01% as calculated by the random adsorption model [16]. It could be then confirmed that O_2_ is photocatalytically generated via pure water splitting. The photocatalytic stability was investigated with Zn-PCN(4.79%). As shown in Fig. S8, no noticeable degradation in photocatalytic activity was observed during the reaction for 72 h, implying that Zn-PCN is very stable for water splitting reactions. The almost unchanged XANES, XPS and FT-EXAFS spectra before and after the long-term photocatalytic reaction (Fig. S9) further demonstrate the good stability of Zn-PCN for overall water splitting.

### Mechanism Analysis for Photocatalytic Overall Water Splitting

**Fig. 6:**
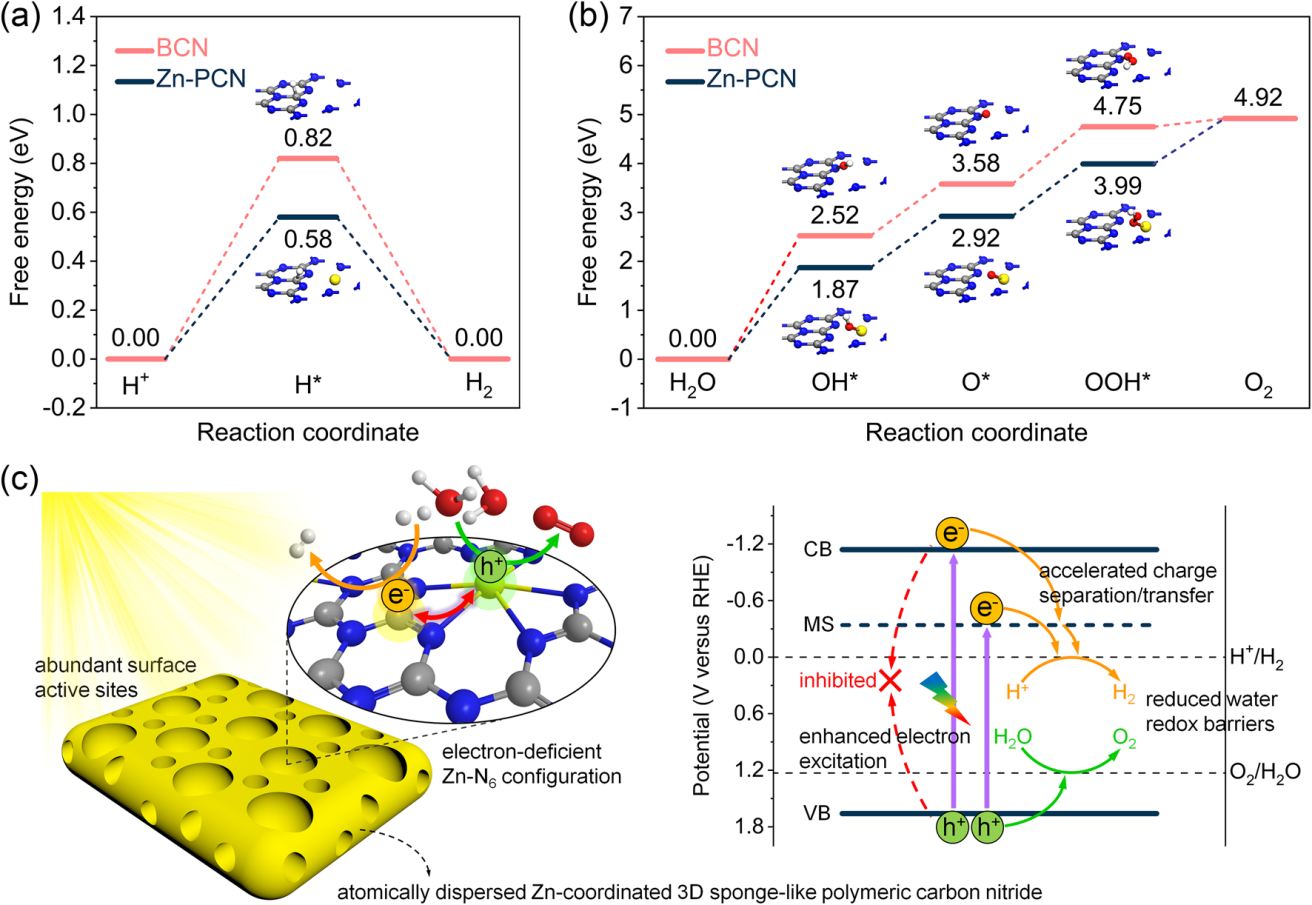
Calculated free energy diagrams of **a** water reduction to H_2_ and **b** water oxidation to O_2_ for BCN and Zn-PCN. **c** Proposed reaction mechanism of photocatalytic overall water splitting into H_2_ and O_2_ over Zn-PCN. The gray, blue, yellow, red and white spheres represent the C, N, Zn, O and H atoms, respectively

All the above experimental observations and theoretical calculations preliminarily infer that the enhanced photocatalytic performance achieved for Zn-PCN should be attributed to the Zn single-atom modulated electronic configuration enabling promoted electron excitation and accelerated charge separation/transfer, as well as the abundant surface active sites derived from the 3D porous structure for accelerated surface redox reactions. To deepen the insights into the underlying fundamentals determinative to the improved photocatalytic activity, water reduction and oxidation reaction paths were theoretically calculated for BCN and Zn-PCN, with free energy diagrams shown in Fig. 6a, b. For BCN, an energy difference of 0.82 eV is required for the formation of H* at C atoms for H_2_ evolution (Fig. 6a). By comparison, Zn-PCN holds a much smaller energy difference of 0.58 eV (Fig. 6a), meaning the lower energy barrier for H* formation [41, 48]. As illustrated in the differential charge density calculations (Fig. 3h), rather than N atoms in BCN, the introduced Zn single atoms with an electron-deficient state are favorable to act as water oxidation active sites in Zn-PCN. For O_2_ evolution, Zn-PCN also has lower energy barriers than BCN from H_2_O adsorption to intermediates (*i.e.*, OH*, O* and OOH*) formation (Fig. 6b). These DFT calculation results indicate that water splitting into H_2_ and O_2_ at Zn-PCN requires lower energy than at BCN, with more electrons and holes accumulated at the water reduction and oxidation active sites, respectively, leading to the enhanced adsorption and activization of H_2_O molecules and then the reduced energy barriers for the formation of H* and OH* intermediates.

On the basis of the comprehensive analysis above in electronic structures, band structures, photoexcitation and charge transfer dynamics as well as water redox reaction paths, a possible mechanism for the improved photocatalytic activity over Zn-PCN is shown in Fig. 6c. Along with the 3D porous structure beneficial to abundant surface active sites for water splitting, the anchored Zn single atoms featured with an electron-deficient Zn-N_6_ configuration effectively modulate the electronic configuration of Zn-PCN, which can induce a midgap energy level, increase the carrier concentration, strengthen the charge polarization in Zn-PCN and redistribute the charges with electrons and holes enriched at the water redox active sites, contributing to promoted electron excitation, accelerated charge separation and transfer as well as reduced overpotentials of water redox reactions, finally enabling Zn-PCN to realize visible-light photocatalytic pure water splitting with H_2_ and O_2_ stoichiometrically evolved.

## Conclusion

In this work, atomically dispersed Zn-anchored 3D sponge-like PCN (Zn-PCN) characteristic of a unique Zn-N_6_ electron-deficient configuration was successfully synthesized via an intermediate coordination strategy. The obtained Zn-PCN achieves significantly improved photocatalytic H_2_ production activity than BCN from water splitting in the presence of sacrificial agent, as well as realizes photocatalytic overall water splitting to stoichiometrically produce H_2_ and O_2_ with good durability under visible light. It is experimentally and theoretically revealed that such excellent photocatalytic water splitting performance should be benefitted from the modulated electronic configuration of PCN by the anchored Zn single atoms, which can induce a midgap energy level, increase the carrier concentration and strengthen the charge polarization, resulting in enhanced electron excitation, accelerated charge separation/transfer and reduced water redox barriers. In addition, the 3D porous structure would provide abundant surface reactive sites for water splitting. This work reports a novel single-atom photocatalyst for water splitting, and deepens the understanding of structure–activity relationship from the viewpoint of atomic coordination and electronic configuration, which is believed to provide informative principles for the design of efficient photocatalysts toward solar energy conversion.

## Supplementary Information

Below is the link to the electronic supplementary material.

Supplementary file1 (PDF 1443 KB)

## References

[1] Bard AJ, Fox MA (1995). Artificial photosynthesis: solar splitting of water to hydrogen and oxygen. Acc. Chem. Res..

[2] Chen X, Shen S, Guo L, Mao SS (2010). Semiconductor-based photocatalytic hydrogen generation. Chem. Rev..

[3] Tan HL, Abdi FF, Ng YH (2019). Heterogeneous photocatalysts: an overview of classic and modern approaches for optical, electronic, and charge dynamics evaluation. Chem. Soc. Rev..

[4] Bie C, Wang L, Yu J (2022). Challenges for photocatalytic overall water splitting. Chem.

[5] Zhang G, Lan ZA, Wang X (2016). Conjugated polymers: catalysts for photocatalytic hydrogen evolution. Angew. Chem. Int. Ed..

[6] Wang L, Zhang Y, Chen L, Xu H, Xiong Y (2018). 2D polymers as emerging materials for photocatalytic overall water splitting. Adv. Mater..

[7] Thangamuthu M, Ruan Q, Ohemeng PO, Luo B, Jing D (2022). Polymer photoelectrodes for solar fuel production: progress and challenges. Chem. Rev..

[8] Ong WJ, Tan LL, Ng YH, Yong ST, Chai SP (2016). Graphitic carbon nitride (g-C_3_N_4_)-based photocatalysts for artificial photosynthesis and environmental remediation: are we a step closer to achieving sustainability?. Chem. Rev..

[9] Li Y, Kong T, Shen S (2019). Artificial photosynthesis with polymeric carbon nitride: when meeting metal nanoparticles, single atoms, and molecular complexes. Small.

[10] Zhang Q, Liu X, Chaker M, Ma D (2021). Advancing graphitic carbon nitride-based photocatalysts toward broadband solar energy harvesting. ACS Mater. Lett..

[11] Zhao D, Guan X, Shen S (2022). Design of polymeric carbon nitride-based heterojunctions for photocatalytic water splitting: a review. Environ. Chem. Lett..

[12] Wang X, Maeda K, Thomas A, Takanabe K, Xin G (2009). A metal-free polymeric photocatalyst for hydrogen production from water under visible light. Nat. Mater..

[13] Li B, Si Y, Fang Q, Shi Y, Huang WQ (2020). Hierarchical self-assembly of well-defined louver-like P-doped carbon nitride nanowire arrays with highly efficient hydrogen evolution. Nano-Micro Lett..

[14] Xue F, Si Y, Wang M, Liu M, Guo L (2019). Toward efficient photocatalytic pure water splitting for simultaneous H_2_ and H_2_O_2_ production. Nano Energy.

[15] Chen X, Shi R, Chen Q, Zhang Z, Jiang W (2019). Three-dimensional porous g-C_3_N_4_ for highly efficient photocatalytic overall water splitting. Nano Energy.

[16] Zhao D, Wang Y, Dong CL, Huang YC, Chen J (2021). Boron-doped nitrogen-deficient carbon nitride-based Z-scheme heterostructures for photocatalytic overall water splitting. Nat. Energy.

[17] Chen X, Wang J, Chai Y, Zhang Z, Zhu Y (2021). Efficient photocatalytic overall water splitting induced by the giant internal electric field of a g-C_3_N_4_/rGO/PDIP Z-scheme heterojunction. Adv. Mater..

[18] Zhang Q, Chen X, Yang Z, Yu T, Liu L (2022). Precisely tailoring nitrogen defects in carbon nitride for efficient photocatalytic overall water splitting. ACS Appl. Mater. Interf..

[19] Wang B, Cai H, Shen S (2019). Single metal atom photocatalysis. Small. Methods.

[20] Pu Z, Amiinu IS, Cheng R, Wang P, Zhang C (2020). Single-atom catalysts for electrochemical hydrogen evolution reaction: recent advances and future perspectives. Nano-Micro Lett..

[21] Wei YS, Zhang M, Zou R, Xu Q (2020). Metal-organic framework-based catalysts with single metal sites. Chem. Rev..

[22] Gao C, Low J, Long R, Kong T, Zhu J (2020). Heterogeneous single-atom photocatalysts: fundamentals and applications. Chem. Rev..

[23] Wu X, Zhang H, Zuo S, Dong J, Li Y (2021). Engineering the coordination sphere of isolated active sites to explore the intrinsic activity in single-atom catalysts. Nano-Micro Lett..

[24] Chen Z, Mitchell S, Vorobyeva E, Leary RK, Hauert R (2017). Stabilization of single metal atoms on graphitic carbon nitride. Adv. Funct. Mater..

[25] Zhou P, Li N, Chao Y, Zhang W, Lv F (2019). Thermolysis of noble metal nanoparticles into electron-rich phosphorus-coordinated noble metal single atoms at low temperature. Angew. Chem. Int. Ed..

[26] Zhang L, Long R, Zhang Y, Duan D, Xiong Y (2020). Direct observation for dynamic bond evolution in single-atom Pt/C_3_N_4_ catalysts. Angew. Chem. Int. Ed..

[27] Jin X, Wang R, Zhang L, Si R, Shen M (2020). Electron configuration modulation of nickel single atoms for elevated photocatalytic hydrogen evolution. Angew. Chem. Int. Ed..

[28] Xiao X, Gao Y, Zhang L, Zhang J, Zhang Q (2020). A promoted charge separation/transfer system from Cu single atoms and C_3_N_4_ layers for efficient photocatalysis. Adv. Mater..

[29] Lin Z, Wang Y, Peng Z, Huang YC, Meng F (2022). Single-metal atoms and ultra-small clusters manipulating charge carrier migration in polymeric perylene diimide for efficient photocatalytic oxygen production. Adv. Energy Mater..

[30] Clark SJ, Segall MD, Pickard CJ, Hasnip PJ, Probert MI (2005). First principles methods using CASTEP. Z. Kristallogr. Cryst. Mater..

[31] Li Y, Wang Y, Dong CL, Huang YC, Chen J (2021). Single-atom nickel terminating sp^2^ and sp^3^ nitride in polymeric carbon nitride for visible-light photocatalytic overall water splitting. Chem. Sci..

[32] Zhao D, Chen J, Dong CL, Zhou W, Huang YC (2017). Interlayer interaction in ultrathin nanosheets of graphitic carbon nitride for efficient photocatalytic hydrogen evolution. J. Catal..

[33] Shi L, Chang K, Zhang H, Hai X, Yang L (2016). Drastic enhancement of photocatalytic activities over phosphoric acid protonated porous g-C_3_N_4_ nanosheets under visible light. Small.

[34] Han Q, Wang B, Gao J, Cheng Z, Zhao Y (2016). Atomically thin mesoporous nanomesh of graphitic C_3_N_4_ for high-efficiency photocatalytic hydrogen evolution. ACS Nano.

[35] Liu X, Deng Y, Zheng L, Kesama MR, Tang C (2022). Engineering low-coordination single-atom cobalt on graphitic carbon nitride catalyst for hydrogen evolution. ACS Catal..

[36] Wang J, Xia Y, Zhao H, Wang G, Xiang L (2017). Oxygen defects-mediated Z-scheme charge separation in g-C_3_N_4_/ZnO photocatalysts for enhanced visible-light degradation of 4-chlorophenol and hydrogen evolution. Appl. Catal. B.

[37] Liu C, Qiu Y, Wang F, Wang K, Liang Q (2017). Design of core-shell-structured ZnO/ZnS hybridized with graphite-like C_3_N_4_ for highly efficient photoelectrochemical water splitting. Adv. Mater. Interf..

[38] Yang F, Song P, Liu X, Mei B, Xing W (2018). Highly efficient CO_2_ electroreduction on ZnN_4_-based single-atom catalyst. Angew. Chem. Int. Ed..

[39] Zhang T, Wang F, Yang C, Han X, Liang C (2022). Boosting ORR performance by single atomic divacancy Zn-N_3_C-C_8_ sites on ultrathin N-doped carbon nanosheets. Chem. Catal..

[40] Yu H, Shi R, Zhao Y, Bian T, Zhao Y (2017). Alkali-assisted synthesis of nitrogen deficient graphitic carbon nitride with tunable band structures for efficient visible-light-driven hydrogen evolution. Adv. Mater..

[41] Jiang XH, Zhang LS, Liu HY, Wu DS, Wu FY (2020). Silver single atom in carbon nitride catalyst for highly efficient photocatalytic hydrogen evolution. Angew. Chem. Int. Ed..

[42] Kumar P, Vahidzadeh E, Thakur UK, Kar P, Alam K (2019). C_3_N_5_: a low bandgap semiconductor containing an AZO-linked carbon nitride framework for photocatalytic, photovoltaic and adsorbent applications. J. Am. Chem. Soc..

[43] Chen J, Dong CL, Zhao D, Huang YC, Wang X (2017). Molecular design of polymer heterojunctions for efficient solar-hydrogen conversion. Adv. Mater..

[44] Zhao D, Wang M, Kong T, Shang Y, Du X (2019). Electronic pump boosting photocatalytic hydrogen evolution over graphitic carbon nitride. Mater. Today Chem..

[45] Jiang T, Xie T, Zhang Y, Chen L, Peng L (2010). Photoinduced charge transfer in ZnO/Cu_2_O heterostructure films studied by surface photovoltage technique. Phys. Chem. Chem. Phys..

[46] Seo DB, Trung TN, Kim DO, Duc DV, Hong S (2020). Plasmonic Ag-decorated few-layer MoS_2_ nanosheets vertically grown on graphene for efficient photoelectrochemical water splitting. Nano-Micro Lett..

[47] Lee MG, Yang JW, Park H, Moon CW, Andoshe DM (2022). Crystal facet engineering of TiO_2_ nanostructures for enhancing photoelectrochemical water splitting with BiVO_4_ nanodots. Nano-Micro Lett..

[48] Cai H, Wang B, Xiong L, Bi J, Hao H (2022). Boosting photocatalytic hydrogen evolution of g-C_3_N_4_ catalyst via lowering the Fermi level of co-catalyst. Nano Res..

