# Supplementary material for: Electron-Deficient Zn-N6 Configuration Enabling Polymeric Carbon Nitride for Visible-Light Photocatalytic Overall Water Splitting
Source: Nanomicro Lett. 2022 Nov 14;14:223. doi: 10.1007/s40820-022-00962-x (PMC9663795; doi:10.1007/s40820-022-00962-x)
Supplement: Supplementary file 1 — Supplementary file1 (PDF 1443 KB) [file 40820_2022_962_MOESM1_ESM.pdf]

Supporting Information for

Electron-Deficient Zn-N_6 Configuration Enabling Polymeric Carbon Nitride for Visible-Light Photocatalytic Overall Water Splitting

Daming Zhao[1, 2], Yiqing Wang[1], Chung-Li Dong[3], Fanqi Meng[4], Yu-Cheng Huang[3], Qinghua Zhang[4], Lin Gu[4], Lan Liu[1], Shaohua Shen[1,] *

[1]International Research Center for Renewable Energy, State Key Laboratory of Multiphase Flow in Power Engineering, Xi'an Jiaotong University, Xi'an, 710049, People's Republic of China

[2]State Key Laboratory of Rare Earth Resource Utilization, Changchun Institute of Applied Chemistry, Chinese Academy of Sciences, Changchun, 130022, People's Republic of China

[3]Department of Physics, Tamkang University, New Taipei City, 25137, Taiwan, People's Republic of China

[4]Beijing National Laboratory for Condensed Matter Physics, Institute of Physics, Chinese Academy of Sciences, Beijing, 100190, People's Republic of China

*Corresponding author. E-mail: shshen_xjtu@mail.xjtu.edu.cn (Shaohua Shen)

Supplementary Figures and Tables

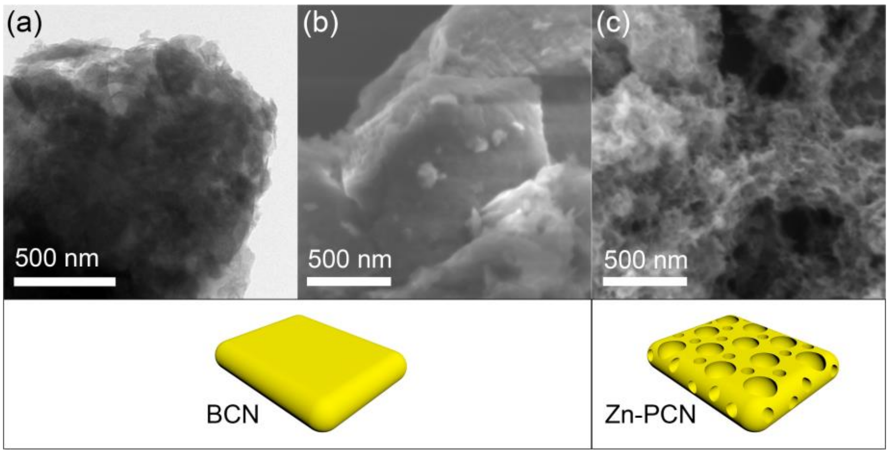

Fig. S1 a TEM and **b** SEM images of BCN. **c** SEM image of Zn-PCN

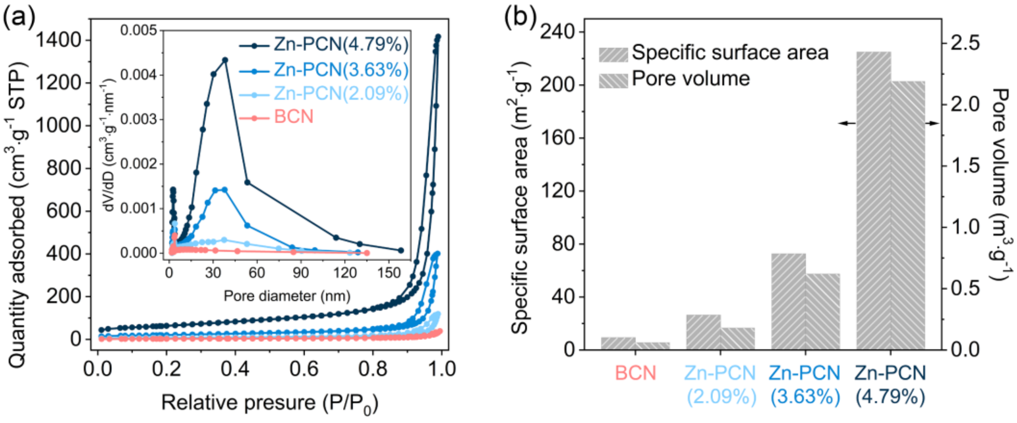

Fig. S2 a N_2 adsorption-desorption isotherms at 77 K and the corresponding pore size distribution curves (inset) of BCN and Zn-PCN. **b** Specific surface area and pore volume of BCN and Zn-PCN

A typical IUPAC type IV adsorption isotherm, commonly regarded as a sign of the mesoporous structure [S1], is observed for Zn-PCN in Fig. S2a. Thus, it could be inferred that Zn-PCN owns a mesoporous structure, and the average pore diameter could be determined to be ~30 nm from the corresponding pore size distribution. With the increasing amounts of $ZnCO_3$ precursor (*i.e.*, Zn loading contents), the adsorption isotherm is steepened and the loop area is increased gradually (Fig. S2a), meaning the increased pore volume. As further shown in Fig. S2b, the pore volume is increased from 0.06 m^3 g^{-1} for BCN to 2.19 m^3 g^{-1} for Zn-PCN(4.79%). Moreover, the specific surface area reaches 224.99 m^2 g^{-1} as high for Zn-PCN(4.79%), which is almost 24 times that for BCN (9.46 m^2 g^{-1}), demonstrating the well developed 3D porous structure obtained by the intermediate coordination strategy.

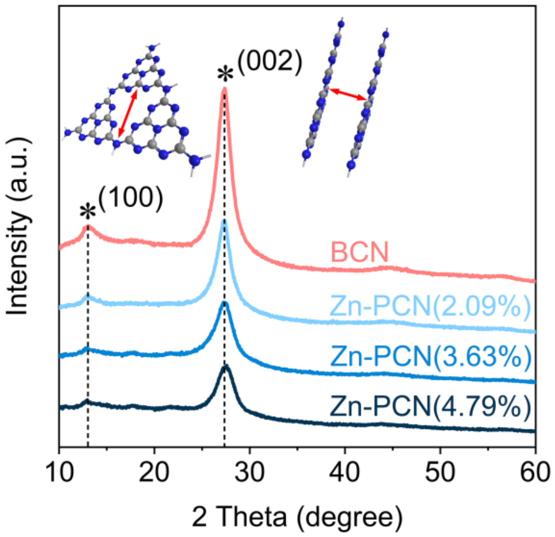

Fig. S3 XRD patterns of BCN and Zn-PCN. The structures of the obtained samples were characterized by XRD patterns

As shown in Fig. S3, BCN displays two feature diffraction peaks at 2θ = 13.1° and 27.3°, which correspond to the (100) plane of the in-plane structural packing of tri-s-triazine units and the (002) plane of interlayer stacking for conjugated aromatic systems, respectively [S2]. Remarkably, both the two peaks show intensities gradually weakened for Zn-PCN with the increasing Zn loading contents, implying the inhibited agglomeration of PCN with the introduction of Zn single atoms through the intermediate coordination strategy [S2].

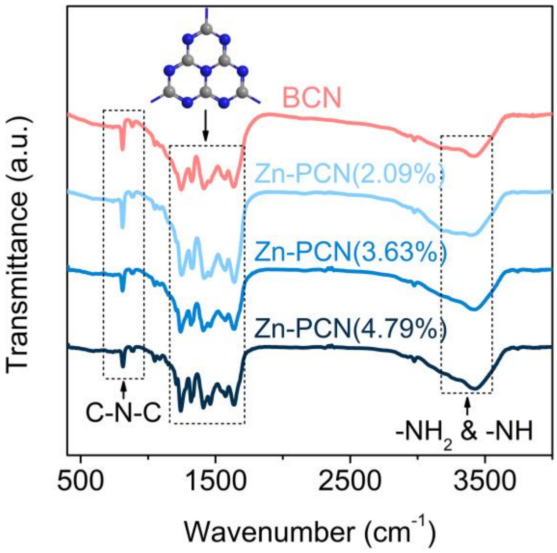

Fig. S4 FTIR spectra of BCN and Zn-PCN. The molecular structures of the as-prepared samples were disclosed by FTIR spectra

As shown in Fig. S4, BCN presents a peak at 810 cm^{-1} typical for the out-of-plane bending mode of tri-s-triazine rings [S3], whilst the peaks locating between 900 and 1800 cm^{-1} originate from the stretching modes of aromatic C-N heterocycles [S3]. Additionally, the multiple broad peaks in the region of 3000-3500 cm^{-1} correspond to the N-H stretching vibrations [S3]. Zn-PCN shows the very similar characteristics in the FTIR spectra as compared to BCN, demonstrating the good retention of both tri-s-triazine ring and trigonal C-N(-C)-C/bridging C-NH-C units of PCN in Zn-PCN.

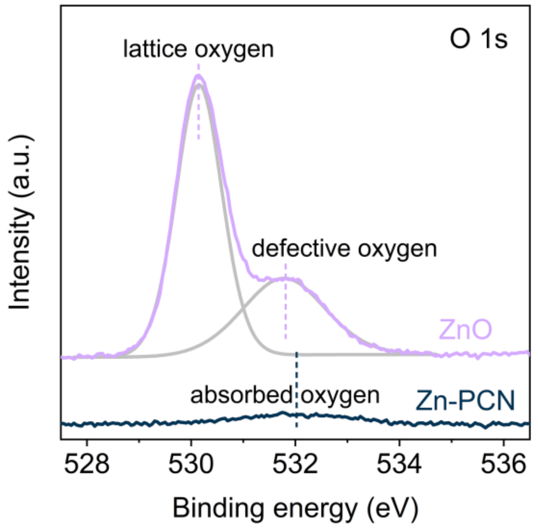

Fig. S5 High-resolution O 1s XPS spectra of Zn-PCN and ZnO

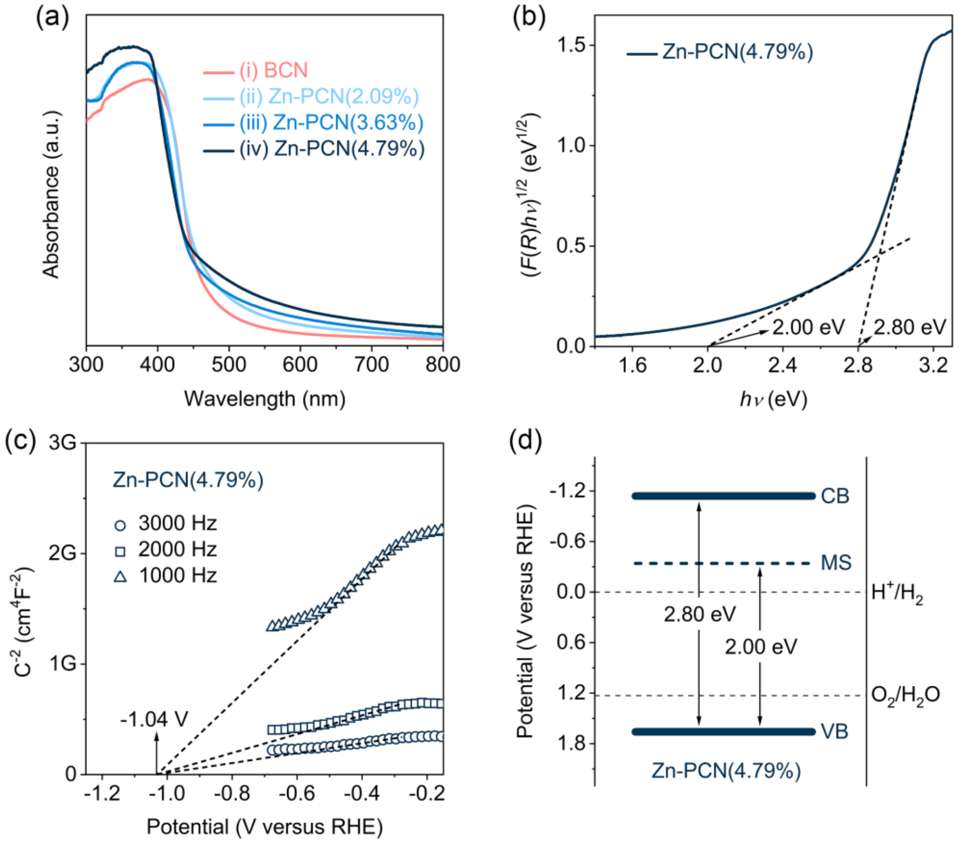

Fig. S6 a Ultraviolet-visible-near infrared diffuse reflectance spectra of CNN and Zn-PCN. **b** Corresponding Tauc plot for Zn-PCN(4.79%) using $(F(R)h\nu)^{1/2}$ (Kubelka-Munk parameter) as a function versus the photon energy. **c** Mott-Schottky plots for Zn-PCN(4.79%). **d** Band structure alignments for Zn-PCN(4.79%)

As determined from the transformed Kubelka-Munk function [S4, S5], the intrinsic bandgap of Zn-PCN(4.79%) is calculated to be 2.80 eV (Fig. S6a-b). Moreover, a bandgap of 2.00 eV, corresponding to the electronic transition from the VB to the midgap state (MS), is identified for Zn-PCN(4.79%) (Fig. S6b). As revealed by the Mott-Schottky plots (Fig. S6c), the flat band potential of Zn-PCN(4.79%) is -1.04 V (versus the reversible hydrogen electrode, RHE). In principle, the CB potential of n-type semiconductor is considered to be ~0.1 eV more negative than that of the flat band potential [S6]. Accordingly, the CB potential for Zn-PCN(4.79%) is -1.14 V (versus RHE). The VB potential then can be calculated to be 1.66 V (versus RHE). Based on these results, Fig. S6d schematically illustrates the band structures of Zn-PCN(4.79%) with the water splitting redox potential as a reference. Obviously, the band structures of Zn-PCN(4.79%) could satisfy the thermodynamic requirement for photocatalytic water splitting into H_2 and O_2.

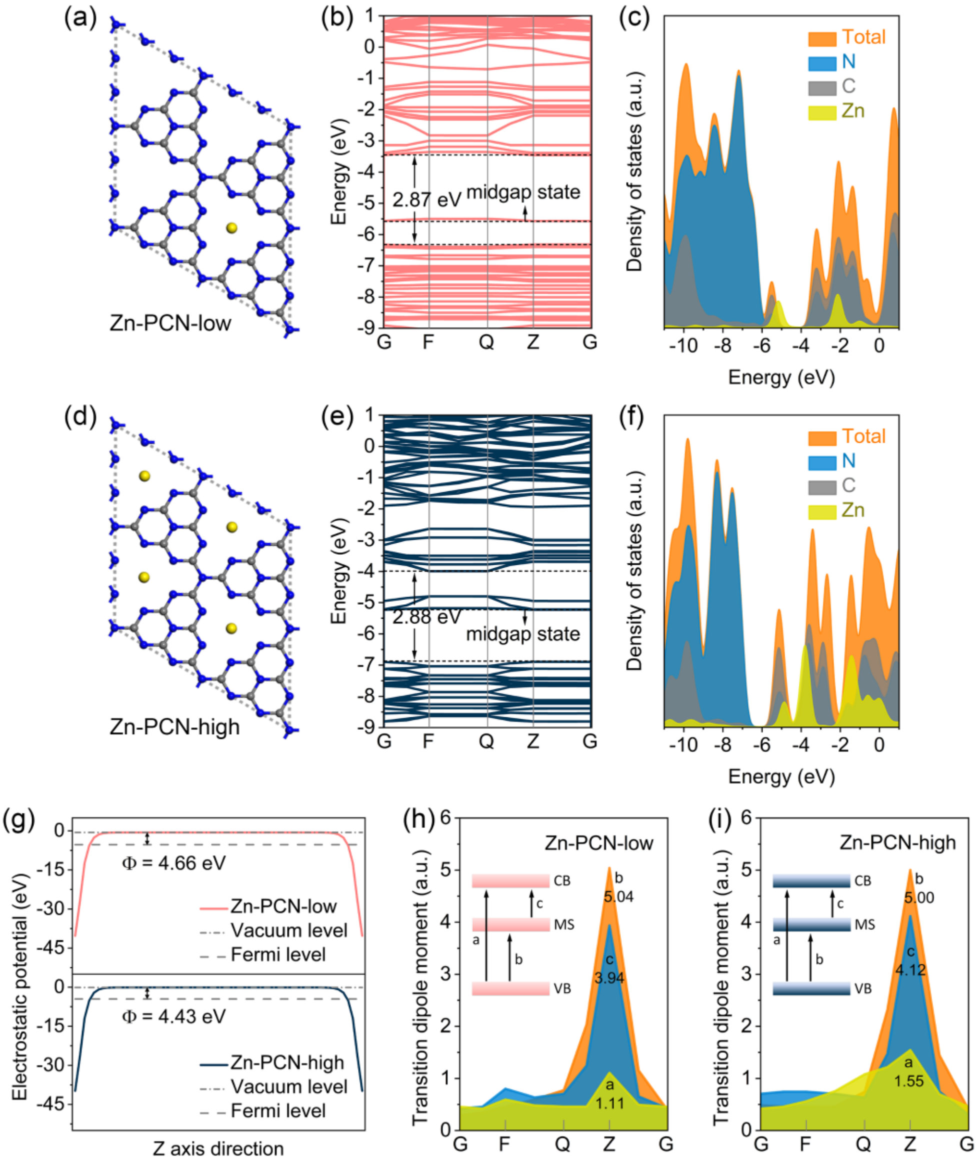

Fig. S7 Structure models of **a** Zn-PCN-low and **d** Zn-PCN-high. The gray, blue, and yellow spheres represent the C, N, and Zn atoms, respectively. **b**,**e** Calculated band structures and **c**,**f** corresponding DOS of Zn-PCN-low and Zn-PCN-high, respectively. **g** Calculated work functions of Zn-PCN-low and Zn-PCN-high. **h**,**i** Transition dipole moments for Zn-PCN-low and Zn-PCN-high, respectively. The insets in **h**,**i** illustrate the different transition paths

Theoretical calculations for PCN coordinated with different loading contents of Zn single atoms (*i.e.*, Zn-PCN-low and Zn-PCN-high) were carried out. As shown in Fig. S7a-g, with bandgap

keeping almost unchanged, both the CB and VB energy levels are negatively shifted, the Zn-related midgap state (MS) energy level is positively shifted, and work function (Φ) is reduced for Zn-PCN-high, as compared to Zn-PCN-low. It is notable that the transition dipole moment related to the intrinsic excitation (transition path a) of Zn-PCN-high is increased as compared to that of Zn-PCN-low (Fig. S7h-i), implying that the more Zn single atoms would contribute to the stronger electron excitation in Zn-PCN [S7, S8].

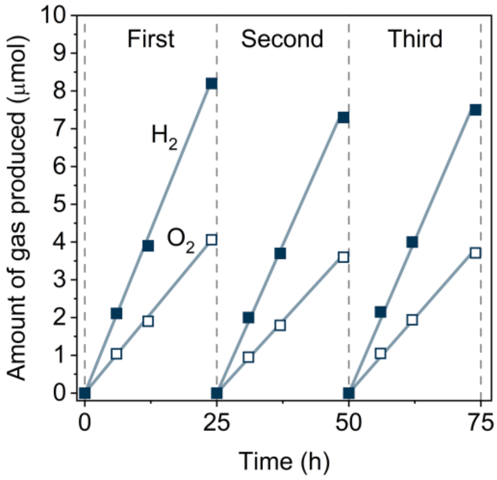

Fig. S8 Cycling of photocatalytic overall water splitting over Zn-PCN(4.79%), where each cycle was started with a one-hour argon flush

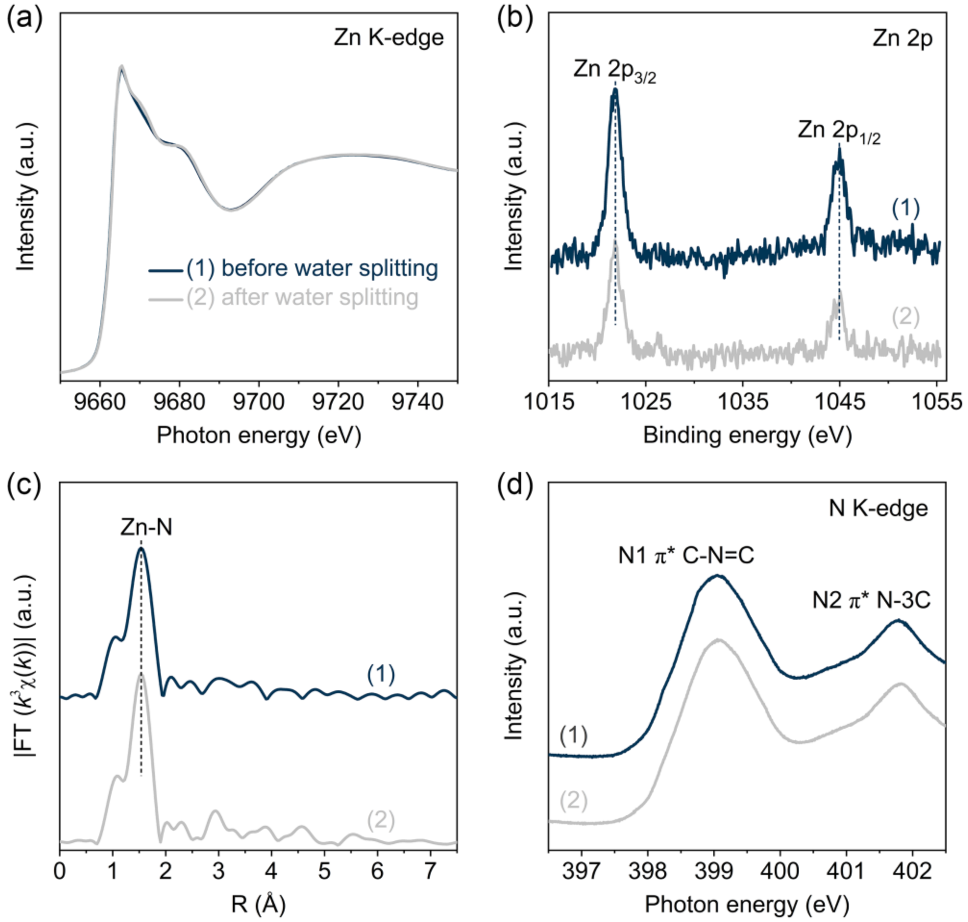

Fig. S9 **a** Zn K-edge XANES spectra, **b** high-resolution Zn 2p XPS spectra, **c** Zn K-edge k^3-weighted FT-EXAFS spectra and **d** N K-edge XANES spectra of Zn-PCN (1) before and (2) after photocatalytic overall water splitting reaction

Table S1 Specific surface area, pore volume and pore size of BCN and Zn-PCN

Samples	Specific surface area ($m^2 \cdot g^{-1}$)	Pore volume ($cm^3 \cdot g^{-1}$)	Pore size (nm)
BCN	9.46	0.06	20.38
Zn-PCN(2.09%)	26.39	0.18	24.36
Zn-PCN(3.63%)	72.66	0.62	29.29
Zn-PCN(4.79%)	224.99	2.19	33.80

Table S2 Structural parameters extracted from the Zn K-edge FT-EXAFS fitting

Samples	Path	Coordination number	R (Å)	σ^2 (10^{-3}Å^2)	ΔE_0 (eV)
Zn foil	Zn-Zn	6.000	2.542	1.268	-1.402
Zn-PCN	Zn-N	5.824	1.995	0.7260	1.580

Table S3 Kinetic parameters of the emission decay analysis of BCN and Zn-PCN

Samples	τ_1 (ns)	A_1	τ_2 (ns)	A_2	τ_3 (ns)	A_3	τ_{avg} (ns)	χ^2
BCN	13.5	0.160	7.71	0.178	2.49	0.662	8.48	1.13
Zn-PCN(2.09%)	14.1	0.114	3.62	0.472	1.01	0.414	7.83	1.02
Zn-PCN(3.63%)	12.3	0.171	4.09	0.455	1.58	0.374	7.53	0.997
Zn-PCN(4.79%)	13.9	0.0776	4.30	0.274	1.02	0.648	7.09	0.995

Table S4 Parameters of the EIS analysis of various catalyst electrodes

Samples	R_s (Ω)	R_{ct} (Ω)
BCN	74.16	6143
Zn-PCN(2.09%)	81.51	2591
Zn-PCN(3.63%)	81.28	1688
Zn-PCN(4.79%)	71.05	930

Supplementary References

[S1] S. Shen, D. Zhao, J. Chen, L. Guo, S.S. Mao, Enhanced photocatalytic hydrogen evolution over graphitic carbon nitride modified with Ti-activated mesoporous silica. Appl. Catal. A **521**, 111-117 (2016). http://dx.doi.org/10.1016/j.apcata.2015.11.004

[S2] D. Zhao, J. Chen, C.L. Dong, W. Zhou, Y.C. Huang et al., Interlayer interaction in ultrathin nanosheets of graphitic carbon nitride for efficient photocatalytic hydrogen evolution. J. Catal. **352**, 491-497 (2017). http://dx.doi.org/10.1016/j.jcat.2017.06.020

[S3] Q. Liang, Z. Li, Z.H. Huang, F. Kang, Q.H. Yang, Holey graphitic carbon nitride nanosheets with carbon vacancies for highly improved photocatalytic hydrogen production. Adv. Funct. Mater. **25**(44), 6885-6892 (2015). https://doi.org/10.1002/adfm.201503221

[S4] Y. Zhu, C. Lv, Z. Yin, J. Ren, X. Yang et al., A [001]-oriented Hittorf's phosphorus nanorods/polymeric carbon nitride heterostructure for boosting wide-spectrum-responsive photocatalytic hydrogen evolution from pure water. Angew. Chem. Int. Ed. **59**(2), 868-873 (2020). https://doi.org/10.1002/anie.201911503

[S5] Y. Wei, Z. Wang, J. Su, L. Guo, Metal-free flexible protonated g-C_3N_4/carbon dots photoanode for photoelectrochemical water splitting. ChemElectroChem **5**(19), 2734-2737 (2018). https://doi.org/10.1002/celc.201800550

[S6] M. Wang, S. Xu, Z. Zhou, C.L. Dong, X. Guo et al., Atomically dispersed Janus nickel sites on red phosphorus for photocatalytic overall water splitting. Angew. Chem. Int. Ed. **61**(29), e202204711 (2022). https://doi.org/10.1002/anie.202204711

[S7] Z. Lin, Y. Wang, Z. Peng, Y.C. Huang, F. Meng et al., Single-metal atoms and ultra-small clusters manipulating charge carrier migration in polymeric perylene diimide for efficient photocatalytic oxygen production. Adv. Energy Mater. **12**(26), 2200716 (2022). https://doi.org/10.1002/aenm.202200716

[S8] C. Zhang, Y. Shi, Y. Si, M. Liu, L. Guo et al., Improved carrier lifetime in $BiVO_4$ by spin protection. Nano Lett. **22**(15), 6334-6341 (2022). https://doi.org/10.1021/acs.nanolett.2c02070
